# Correlation between serum high-mobility group box-1 levels and high-sensitivity C-reactive protein and troponin I in patients with coronary artery disease

**DOI:** 10.3892/etm.2013.1095

**Published:** 2013-04-30

**Authors:** HENG-CHEN YAO, AI-PING ZHAO, QIAN-FENG HAN, LEI WU, DAO-KUO YAO, LE-XIN WANG

**Affiliations:** 1 Department of Cardiology, Liaocheng People’s Hospital of Taishan Medical University, Liaocheng, Shandong 252000;; 2 Department of Cardiology, Beijing Friendship Hospital of China Capital Medical University, Beijing 100007, P.R. China;; 3 School of Biomedical Sciences, Charles Sturt University, Wagga Wagga, NSW 2678, Australia

**Keywords:** coronary artery disease, high-mobility group box-1, high-sensitivity C-reactive protein, cardiac troponin I

## Abstract

The aim of this study was to evaluate the correlation between levels of serum high-mobility group box-1 (HMGB1) and high-sensitivity C-reactive protein (hs-CRP) and cardiac troponin I in patients with coronary artery disease. The levels of serum HMGB1, hs-CRP and cardiac troponin I were measured in 98 patients with coronary artery disease and in 30 healthy subjects. The correlation between serum HMGB1 levels and hs-CRP and cardiac troponin I levels was analyzed. Serum HMGB1 levels in patients with coronary artery disease were higher compared with those in healthy volunteers (63.5±15.29 vs. 21.98±4.33 *μ*g/l; P<0.01). Serum HMGB1 levels in patients with acute myocardial infarction were higher compared with those in patients with unstable and stable angina pectoris (77.53±6.86 vs. 63.67±8.6 and 44.39±9.01 *μ*g/l, respectively; both P<0.01). The levels of HMGB1 were positively correlated with hs-CRP and cardiac troponin I levels (r=0.657 and 0.554, respectively; both P<0.01) in patients with coronary artery disease. In conclusion, serum HMGB1 levels were elevated in patients with coronary artery disease, particularly in those with acute myocardial infarction. The levels of HMGB1 were correlated with the levels of hs-CRP and cardiac troponin I.

## Introduction

The inflammatory response plays an important role in cardiac repair following acute myocardial infarction (1,2). Appropriate control of inflammatory responses may contribute to the repair process and promote scar formation (3). High-mobility group box-1 (HMGB1), one of the most important non-histone nuclear proteins, is composed of 215 residues divided into two DNA-binding domains (box A and box B) and a negative C-terminal. HMGB1 is present in the nuclei of eukaryotes, distributed in the liver, brain, spleen, lung, heart, kidney and other vital organs and lymphatic tissue, and participates in a variety of inflammatory processes (4–6). Studies have shown that serum HMGB1 levels are elevated in experimental and clinical acute myocardial infarction, which may indicate an important role in the healing process of the infarcted myocardium (5–9). However, the correlation of serum HMGB1 with other cardiac indices, including high-sensitivity C-reactive protein (hs-CRP) and cardiac troponin I (cTnI), remains unclear.

In the current study, we measured serum HMGB1 levels and determined their correlation with serum hs-CRP and cTnI levels in patients with stable and unstable angina pectoris, as well as acute myocardial infarction.

## Patients and methods

### Patient selection

This study was approved by the Institutional Review Board of Liaocheng People’s Hospital (Liaocheng, China). Ninety-eight consecutive patients admitted to our hospital with coronary artery disease, including stable angina pectoris, unstable angina pectoris and acute myocardial infarction, were enrolled in this study. The diagnosis of stable angina pectoris, unstable angina pectoris and acute myocardial infarction (including ST segment-elevated and non-ST segment-elevated myocardial infarction) was consistent with the current guidelines (10–13). Patients with chronic heart failure, acute infectious diseases, liver and kidney dysfunction, autoimmune diseases and malignant cancer, or immunosuppressive agents or steroid hormone use within 6 months were excluded. Thirty healthy volunteers who had attended the health clinic of our hospital for annual health check-ups were enrolled as the control group. Physical examination, electrocardiogram (ECG), chest X-ray and blood biochemistry examinations revealed no cardiovascular or other chronic diseases. Written informed consent was obtained from the patients or their families prior to the study.

### Transthoracic echocardiography

Echocardiography was conducted as previously described (14). Imaging was performed using a HP 5500 system (Koninklijke Philips Electronics N.V.; Eindhoven, The Netherlands). Patients were imaged in the left lateral decubitus position and the images of the parasternal and apical views (standard long axis, 2- and 4-chamber images) were obtained. The left ventricular ejection fraction (LVEF) was calculated from the conventional apical 2- and 4- chamber images, using the biplane Simpson’s technique (15). Echocardiographic examinations were conducted after admission and on the day of venous blood collection. The cardiologist who performed the echocardiographic studies was not aware of the patient’s HMGB1 or other biochemical test results.

### Biochemical investigations

Fasting venous blood samples were obtained and serum samples were stored at −80°C after centrifuging for 10 min at 167–7 × g on the day after admission. Serum levels of HMGB1 were assessed by enzyme-linked immunosorbent assay (ELISA) according to the manufacturer’s instructions (HMGB-1 ELISA kit; USCN Science Inc., Wuhan, China) (16). The serum hs-CRP and cTnI levels were measured on admission with a high-sensitivity assay in the Central Pathology Laboratory (Liaocheng People’s Hospital, Liaocheng, China). Serum hs-CRP was measured by particle-enhanced immunonephelometry and serum cTnI was measured by ELISA.

### Statistical analysis

Continuous variables are presented as mean ± standard deviation and were compared by a two-tail student’s t-test or one way analysis of variance (ANOVA) for multiple variables. Categorical data are presented as numbers and percentages and were compared with Chi-square test or Fisher’s exact test. Correlations of serum HMGB1 with other biomarkers were assessed by Pearson’s test. All data were analyzed using SPSS 12.0 software (SPSS, Inc., Chicago, IL, USA). A two-sided P-value of ≤0.05 was considered to indicate a statistically significant difference.

## Results

### General findings

A total of 98 patients with confirmed coronary artery disease were included in the study, including 26 patients with stable angina pectoris, 37 with unstable angina pectoris and 35 with acute myocardial infarction. The baseline characteristics of the patients in each group are summarized in Table I. There were no statistically significant differences in age, gender and type of coronary artery disease among the groups (all P>0.05).

### Serum HMGB1 levels

Serum HMGB1 levels in the patients with coronary artery disease were higher compared with those in healthy volunteers (63.5±15.29 vs. 21.98±4.33 *μ*g/l; P<0.01). Serum HMGB1 levels in patients with acute myocardial infarction were higher compared with those in patients with unstable and stable angina pectoris (77.53±6.86 vs. 63.67±8.6 and 44.39±9.01 *μ*g/l, respectively; all P<0.01). Serum HMGB1 levels in patients with unstable angina pectoris and acute myocardial infarction were higher than those in patients with stable angina pectoris (both P<0.01), and serum HMGB1 levels in patients with acute myocardial infarction were higher compared with those in patients with unstable angina pectoris (P<0.01; Table II).

### Correlation between serum HMGB1 and hs-CRP and cTnI

**Figure 1.**
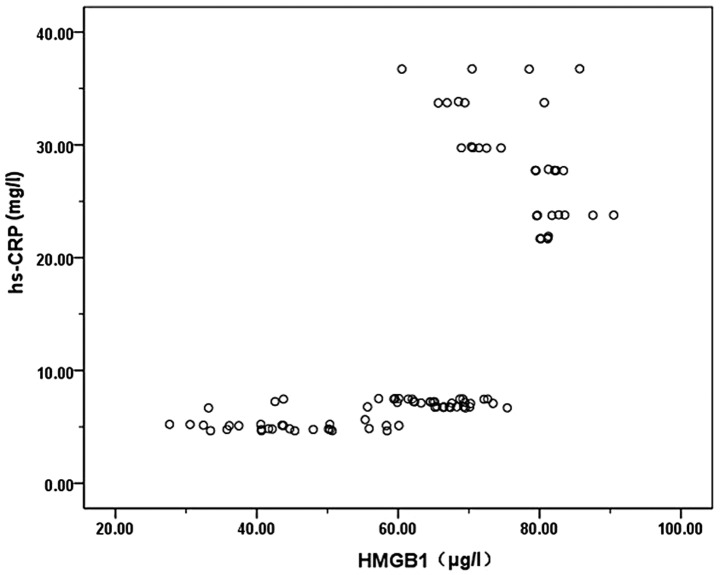
Correlation between serum HMGB1 and hs-CRP. HMGB1, high-mobility group box 1; hs-CRP, high-sensitivity C-reactive protein.

**Figure 2.**
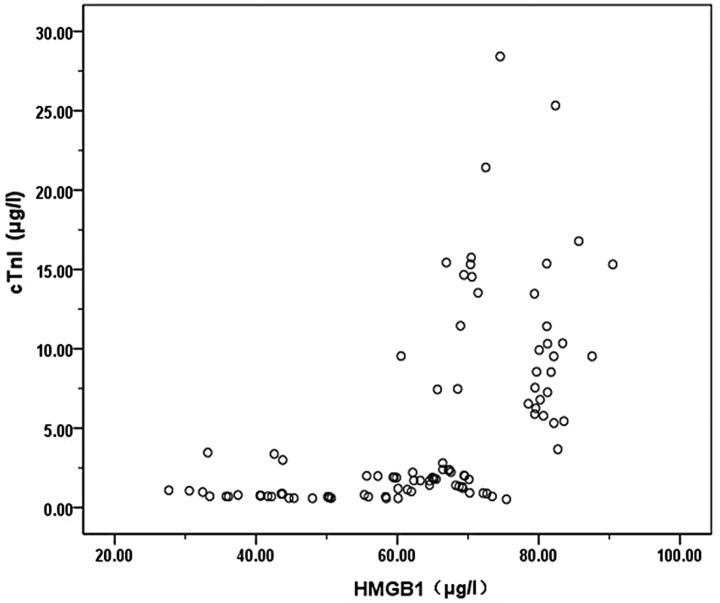
Correlation between serum HMGB1 and cTnI. HMGB1, high-mobility group box 1; c-TnI, cardiac troponin I.

The levels of HMGB1 were positively correlated with those of hs-CRP (r=0.657, P<0.01; Fig. 1) and cTnI (r=0.554, P<0.01; Fig. 2) in patients with coronary artery disease.

## Discussion

The main findings of the current study were that serum HMGB1 levels are elevated in patients with coronary heart disease, particuarly in those with acute myocardial infarction. Furthermore, the levels of HMGB1 are correlated with the levels of hs-CRP and cTnI. These findings suggest that serum HMGB1 may be an alternative predictor of disease severity in patients with coronary artery disease.

Inflammation causes coronary atherosclerotic plaque instability and leads to the activation of endothelial cells, which results in plaque rupture and the stimulation of thrombosis and myocardial ischemia, as well as myocardial infarction (17). Serum hs-CRP levels may increase in the process of inflammation of the myocardium and higher levels of hs-CRP are correlated with the severity of the inflammation of the injured myocardium (17). The levels of cTnI, which is released in the infarcted area of the myocardium, are often elevated in acute myocardial infarction.

The high-mobility group of proteins belong to a class of nuclear non-histone proteins with three families: A, B and N. HMGB1 is a member of the B family of high mobility group proteins, and is mainly located in cell nuclei. A previous study identified that HMGB1 is released and transferred to the outside of the cell following the induction of lipopolysaccharides, tumor necrosis factor-α (TNF-α), interleukin (IL)-1, interferon-γ and biologically active lipids in macrophages, monocytes and dendritic, pituitary, epithelial and liver cells (18). HMGB1 is released passively in necrotic or damaged cells. HMGB1 increases the adhesion of monocytes and the secretion of pro-inflammatory mediators and cytokines, which are involved in the inflammatory response (19).

A previous study demonstrated that left ventricular remodeling and dysfunction may be restricted and survival improved significantly in transgenic mice with a high expression level of HMGB1 4 weeks after the onset of myocardial infarction (9). The formation of capillaries and small arteries was significantly increased, as detected by immunohistochemistry (9). Another study demonstrated that the direct injection of exogenous HMGB1 increases myocardial cell counts in the infarcted myocardial area (4). These results indicate that HMGB1 promotes angiogenesis and improves the functional recovery of the infarcted myocardium, which may be beneficial to myocardial infarction. However, controversy exists as another study has demonstrated that HMGB1 enhances the inflammatory response leading to the deterioration of cardiac function and the occurrence of ventricular remodeling (5). HMGB1 antagonists diminish the harmful results in the injured myocardium (5). Kohno *et al* (8) also showed that a neutralizing anti-HMGB1 antibody effectively weakened the inflammatory reaction following experimental myocardial infarction, which caused a significant reduction in TNF-α and IL-1β levels and macrophage cell count on day 3 in the infarct region, evident thinning and expansion of the ventricular wall in the infarct area and hypertrophy in the non-infarct area, as well as severe left ventricular remodeling. Therefore, it is necessary to clarify the role of HMGB1 in the process and development of various types of coronary artery disease.

A clinical study demonstrated that circulating HMGB1 levels are independently associated with cardiac mortality in ST-segment elevation acute myocardial infarction (6). The serum HMGB1 level on admission was an independent predictor of cardiovascular mortality in unstable angina pectoris and non-ST segment elevation myocardial infarction (6). In patients with ST-segment elevation myocardial infarction undergoing percutaneous intervention treatment, plasma HMGB1 levels were independently associated with mortality in the 10-month follow-up (7). In the present study, we evaluated serum HMGB1 levels in different types of coronary artery disease and identified that serum HMGB1 levels were higher in all types of coronary artery disease than in the controls. The HMGB1 levels in the acute myocardial infarction group were higher than those in the unstable and stable angina pectoris groups. These findings are similar to those in the previously mentioned studies. These results suggest that HMGB1 may be involved in the occurrence and development of acute myocardial infarction and serum HMGB1 levels may be used for evaluating the severity of coronary artery disease.

Our findings indicate that serum HMGB1 levels are positively correlated with hs-CRP levels. This provides evidence for the role of HMGB1 in the inflammatory process of coronary events. A previous study has shown that hs-CRP is correlated with the process and prognosis of coronary artery disease and is an independent predictor of coronary events (17). The current findings suggest that serum HMGB1 may be used for evaluation of severity and stratification of coronary artery disease.

Additionally, we identified that serum HMGB1 is positively correlated with cTnI in patients with coronary artery disease. This result suggests that serum HMGB1 may also be an indicator of necrosis of the myocardium and may be used for evaluation of severity and stratification in this setting, particularly in acute myocardial infarction.

One limitation of our study was the lack of follow-up data in the study groups, which limited the value of serum HMGB1 in risk stratification and prognosis in patients with coronary artery disease. However, our preliminary results demonstrated significant positive results, which suggest a role of HMGB1 in coronary artery disease.

In conclusion, serum HMGB1 levels are elevated in patients with coronary artery disease, particularly in those with acute myocardial infarction. The serum levels of HMGB1 are correlated with the levels of hs-CRP and cTnI. Further studies are required to ascertain the predictive value of serum HMGB1 in patients with coronary artery disease.

## Figures and Tables

**Table I.** Baseline characteristics of the study groups.

Variables	Coronary artery disease			Control (n=30)

	SAP (n=26)	UAP (n=37)	AMI (n=35)	
Male (%)	14 (53.8)^a^	21 (56.8)^a^	20 (57.1)^a^	16 (53.3)
Age (year)	53.46±11.56^a^	58.81±12.35^a^	58.43±12.93^a^	53.43±8.72
Hypertension (%)	16 (61.5)^b^	14 (37.8)^b^	21 (60.0)^b^	9 (30.0)
Diabetes (%)	8 (30.8)^b^	10 (27.0)^a^	14 (40.0)^c^	2 (6.7)
Smoking (%)	10 (38.5)^a^	15 (40.5)^b^	18 (51.4)^c^	5 (16.7)
Drinking (%)	4 (15.4)^a^	7 (18.9)^a^	7 (20.0)^a^	5 (16.7)
ACEI (%)	13 (50.0)^b^	12 (32.4)^a^	15 (42.9)^b^	6 (20.0)
LVEDD (mm)	48.12±6.45^b^	48.38±5.86^b^	51.22±6.00^c^	44.50±5.61
LVEF (%)	60.85±6.41^c^	58.35±5.99^c^	55.57±6.20^c^	59.20±5.74

Data are presented as mean ± standard deviation (SD) or n (%). ACEI, angiotensin-converting enzyme inhibitor; LVEDD, left ventricular end diastolic diameter; LVEF, left ventricular ejection fraction; SAP, stable angina pectoris; UAP, unstable angina pectoris; AMI, acute mycardial infarction.

a P>0.05 vs. control;

b P<0.05 vs. control;

c P<0.01 vs. control

**Table II.** Biochemical variables in the study groups.

Variables	Coronary artery disease				Control (n=30)

	Total (n=98)	SAP (n=26)	UAP (n=37)	AMI (n=35)	
HMGB1 (*μ*g/l)	63.50±15.29^a^	44.39±9.01^a^	63.67±8.60^a,b^	77.53±6.86^a–c^	21.98±4.33
hs-CRP (mg/l)	14.11±11.06^a^	4.95±0.25^a^	7.11±0.31^a,b^	28.32±4.87^a–c^	2.11±0.19
c-TnI (*μ*g/l)	4.95±5.92^a^	0.73±0.14	1.78±0.69^a^	11.43±5.65^a–c^	0.07±0.09

Data are presented as mean ± standard deviation (SD). HMGB1, high-mobility group box 1; hs-CRP, high-sensitivity C-reactive protein; cTnI, cardiac troponin I; SAP, stable angina pectoris; UAP, unstable angina pectoris; AMI, acute mycardial infarction.

a P<0.01 vs. control;

b P<0.01 vs. SAP group;

c P<0.01 vs. UAP group.
